# High prevalence of β-lactam and fluoroquinolone resistance in various phylotypes of *Escherichia coli* isolates from urinary tract infections in Jiroft city, Iran

**DOI:** 10.1186/s12866-023-02860-7

**Published:** 2023-04-22

**Authors:** Saleh Afsharikhah, Reza Ghanbarpour, Parvin Mohseni, Nasrin Adib, Mahboube Bagheri, Maziar Jajarmi

**Affiliations:** 1 Department of Pathobiology, Faculty of Veterinary Medicine, Shahid Bahonar University of Kerman, Kerman, Iran; 2 Molecular Microbiology Research Group, Shahid Bahonar University of Kerman, Kerman, Iran; 3 Department of Food Science and Technology, Bardsir Faculty of Agriculture, Shahid Bahonar University of Kerman, Kerman, Iran

**Keywords:** *Escherichia coli*, Antibiotic resistance, UTI

## Abstract

**Background:**

Urinary tract infection (UTI) is one of the most prevalent infectious diseases with worldwide health threatening. Antimicrobial resistant strains of *Escherichia coli* (*E. coli*) are a common cause of UTI which were identified as a treatment challenge. This study aimed to assay the prevalence of common β-lactam resistance genes including *bla*_TEM_, *bla*_SHV_, *bla*_CTX-M_ and *bla*_CMY_ and phenotypic resistance to commonly used β-lactam and fluoroquinolone antibiotics in UTIs. These factors were evaluated in various phylogenetic groups (phylotypes) of *E. coli* isolates. Real-time PCR was applied to detect β-lactam resistance genes and conventional PCR was used to determine the phylotypes. Phenotypic resistance against β-lactams (ceftazidime, cefotaxime, aztreonam and ceftriaxone) and fluoroquinolones (ciprofloxacin) were identified by the disc diffusion technique. The ability of extended spectrum β-lactamases (ESBLs) production in *E. coli* isolates was detected using the combined disc diffusion method.

**Results:**

The prevalence of resistance genes were 89.6% for *bla*_TEM_, 44.3% for *bla*_CTX-M_, 6.6% for *bla*_SHV_ and 0.9% for *bla*_CMY_. The two high prevalent phylotypes were B2 (29.2%) and D (17.9%) followed by E (14.1%), F (9.4%), C (6.6%) and 10.3% of isolates were unknown in phylotyping. Disc diffusion results showed high prevalence of antibiotic resistance to cefotaxime (88.6%), aztreonam (83%), ceftireaxon (77.3%), ceftazidime (76.4%) and ciprofloxacin (55.6%). Totally, 52.8% of isolates were found as phenotypical ESBL-producers.

**Conclusions:**

This study’s results confirmed an explosion of antibiotic resistance amongst *E. coli* isolates from UTI against β-lactams and fluoroquinolones. Findings explain the necessity of deep changes in quantity and quality of drug resistance diagnosis and antibiotic therapy strategies. More studies are suggested to better and confident evaluations.

## Background

Urinary tract infections (UTIs) are amongst the most common infectious diseases, especially in women, caused by different microorganisms, such as *Escherichia coli* (*E. coli*), *Enterococcus*, *Staphylococcus, Proteus*, *Klebsiella*, *Pseudomonas*, etc. [1]. Uropathogenic *E. coli* (UPEC) pathotype causes more than the three-quarters of UTIs via several virulence factors such as adhesins, capsule, siderophores and toxins [2–4].

Several antibiotics are considered for treatment of cystitis and pyelonephritis, including oral β-lactams (amoxicillin–clavulanic acid or third-generation cephalosporin), fluoroquinolones (ciprofloxacin or levofloxacin), nitrofurantoin, fosfomycin or trimethoprim–sulfamethoxazole [5]. It is a public health issue that the widespread use of antibiotics for the treatment of UTIs has led to the growth of antimicrobial resistant UPECs, which makes it harder to treat, prevent, and manage UTIs.

Resistance against β-lactam agents can occur via (i) mutation or expression of alternative penicillin-binding proteins (PBPs) as the drug target, (ii) downregulation of porins to reduce the bacterial permeability against β-lactams, (iii) over-expression of efflux systems which are membrane transport proteins to export drug substrates and (iv) production of β-lactamases that hydrolyze the β-lactam amide [6]. The production of extended spectrum β-lactamases (ESBLs) is a primary β-lactam resistance mechanism in Gram-negative bacteria. There are four distinct classes of ESBLs, termed A, B C and D based on specific sequence motifs and hydrolytic mechanism in Ambler classification system. Some main enzyme families are TEM, SHV, CTX-M and KPC in class A; NDM and VIM in class B; CMY and ADC in class C; and OXA in Class D [6].

Resistance against the quinolones/fluoroquinolones can occur via three mechanisms; (i) chromosomal mutations that change the targets of the drug, such as GyrA subunits of DNA gyrase and ParC of topoisomerase IV, (ii) mutations related to reduce the drug concentration in bacterial cytoplasm via over-expression of efflux pumps and downregulation of porins, and (iii) PMQR (plasmid-mediated quinolone resistance) genes, such as *qnr* gene responsible to proteins that protect DNA gyrase or topoisomerase IV, *aac(6′)-lb-cr* gene acetylating quinolones and *qepA* and *oqxAB* genes which increase the outflow of the drug molecules through efflux pumps [7].

Antimicrobial resistance genes (ARGs) could be shared among *E. coli* strains via mobile genetic elements, including conjugative plasmids, transposons, insertion sequences and genomic islands [8]. Transmission of these elements leads to recombination in *E. coli* strains in a moderate level. Nevertheless, *E. coli* populations are clonal and could be classified to various phylogenetic groups [9]. Based on presence or absence of four genetic sequences named *chuA*, *yjaA*, *TspE4* and *arpA*, *E. coli* could be categorized into the phylotypes A, B1, B2, D, C, E, F, G and *Escherichia* cryptic clade I [10].

In this study, the phenotypic antibiotic resistance against β-lactams and fluoroquinolones was first studied in* E. coli* isolates from women UTIs in Jiroft city of Iran. Then some of the most important β-lactam resistance genes were screened in the isolates. Finally, phylotypes were determined, and all variables were analyzed in relation to each other.

## Methods

### Sampling, E. coli isolation and confirmation

In this study, urine samples were collected in two laboratories in Jiroft city (southeast of Iran) during the spring season of 2021. The total number of UTI referrals to the laboratories was 168 cases. All urine samples belonged to non-hospitalized women with uncomplicated UTI in Jiroft; they were premenopausal and non-pregnant women without urinary tract abnormality which mostly showed acute cystitis and pyelonephritis in clinical examinations by physicians. The women with suspected UTI submitted their samples to medical diagnostic laboratories in Jiroft for microbial examination.

All sampling procedures were done in the medical diagnostic laboratories; the patients washed their hands before collecting the sample. They collected a midstream urine sample in sterile containers without touching the inside of it. Finally, they closed the container and delivered to the laboratory for next steps.

The urine samples were cultured onto MacConkey agar plates and were incubated at 37 °C for 24–48 h. Among 168 urine samples, 106 (63%) MacConkey agar plates showed suspected *E. coli* colonies. One single smooth and pink colony was selected from each plate and confirmed by IMViC biochemical technique including indole, methyl red, Voges-Proskauer and citrate tests [11].

### Phenotypic assessment of E. coli strains

In this study, the antimicrobial resistance was identified using the Kirby Bauer disk diffusion method; the antibiotics were ceftazidime (CAZ; 30 µg), cefotaxime (CTX; 30 µg), cefotzxime clavulanate (CZA; 30 µg), ceftazidime clavulanate (CTC; 30 µg), aztreonam (AZT; 30 µg), ciprofloxacin (CP; 5 μg) and ceftriaxone (CRO; 30 µg). As a test control, *E. coli* ATCC 25,922 was utilized, and the findings were evaluated according to the Institute of Clinical and Laboratory Standards Institute (CLSI 2018; Table 2). Also, the *E. coli* strains with a ≥ 5-mm increase in zone diameter for cefotaxime-clavulanate vs the zone diameter of cefotaxime or a ≥ 5-mm increase in zone diameter for ceftazidime-clavulanate vs the zone diameter of ceftazidime were considered as ESBL-producing *E. coli* strains [12].

### DNA extraction

Total genomic DNA of the confirmed *E. coli* strains was extracted by boiling technique; a single colony from each sample was suspended in 400 µL sterile distilled water and heated at 98–100 °C in a heating block (Eppendorf, Germany) for 10–15 min. Then, lysates were centrifuged (13,000 × g, 2 min), and the supernatants were moved to a new microtube and stored at -20 °C as DNA templates for next steps [13].

### Real-time and conventional PCR for β-lactamase genes

Four antimicrobial resistance genes including *bla*_TEM_, *bla*_SHV_, *bla*_CMY_ and *bla*_CTX-M_, were screened using Real-time polymerase chain reaction (PCR) and the positive samples were reconfirmed by conventional PCR. For Real-time PCR step, the reactions were uniplex and arranged in 25 µL volume for each gene including 2 µL DNA extract, 0.4 µM from each primer [14], 12.5 µL RealQ Plus 2 × Master Mix Green (Ampliqon, Denmark) and distilled water up to volume of reaction. Thermal cycler program was included 95 °C for 15 min followed by 30 cycles of 95 °C for 15 s; 50 °C for 15 s and 70 °C for 20 s. Positive controls were *Klebsiella* ATCC 700,603 (for *bla*_CTX-M_), *E*. *coli* ATCC 35,218 (for *bla*_TEM_ and *bla*_SHV_) and we didn’t have positive control for *bla*_CMY_. One *E. coli* strain (without the four resistance genes) was used to negative control. The real-time PCR was done via LightCycler® 96 System (Roche Diagnostics GmbH, Mannheim, Germany).

For more confirmation of the results in previous step, conventional PCR were performed on the positive samples. Uniplex PCR were carried out in 25 µL reactions containing 12.5 µL 2X Taq PCR Master Mix (pars tous, Iran), 0.4 µM of each forward and reverse primer [14], 8 µL of sterile water and 2.5 µL of extracted bacterial DNA. The PCR steps were initial denaturation (95 °C for 10 min), 35 thermal cycles including denaturation (95 °C for 30 s), annealing [55 °C for 30 s (for *bla*_TEM_ and *bla*_SHV_) and 60 °C for 30 s (for *bla*_CTX-M_ and *bla*_CMY_)] and elongation (72 °C for 1 min). Finally, an elongation step was attached at 72 °C for 5 min.

### PCR for phylogenetic classification

In this study, the *E. coli* strains were phylotyped by Clermont et al. (2013) scheme using a quadriplex PCR [10]. The sequences *arpA* (400 bp)*, chuA* (288 bp)*, yjaA* (211 bp) and TspE4.C2 (152 bp) were targeted to determine phylotypes including A, B1, B2, C, D, E, F and cryptic clade I. The strain EcoR62 was used as positive control in PCR examinations. Samples were subjected to a 35 PCR cycles including 10 s denaturation at 94 °C, 25 s annealing at 59 °C, and 5 s elongation at 72 °C. The PCR products were electrophoresed on 1.3% agarose gel for 60 min at 80 V. The electrophoresed gel was analyzed by gel documentation imaging system (vilber lourmat, France).

All data related to the presence or absence of phylogenetic groups and antibiotic resistance in each isolate were entered into Excel (Microsoft 2016) and SPSS (SPSS 24; IBM) programs to calculate the prevalence percentage in descriptive statistics.

## Results

### Phenotypic antimicrobial resistance

**Fig. 1:**
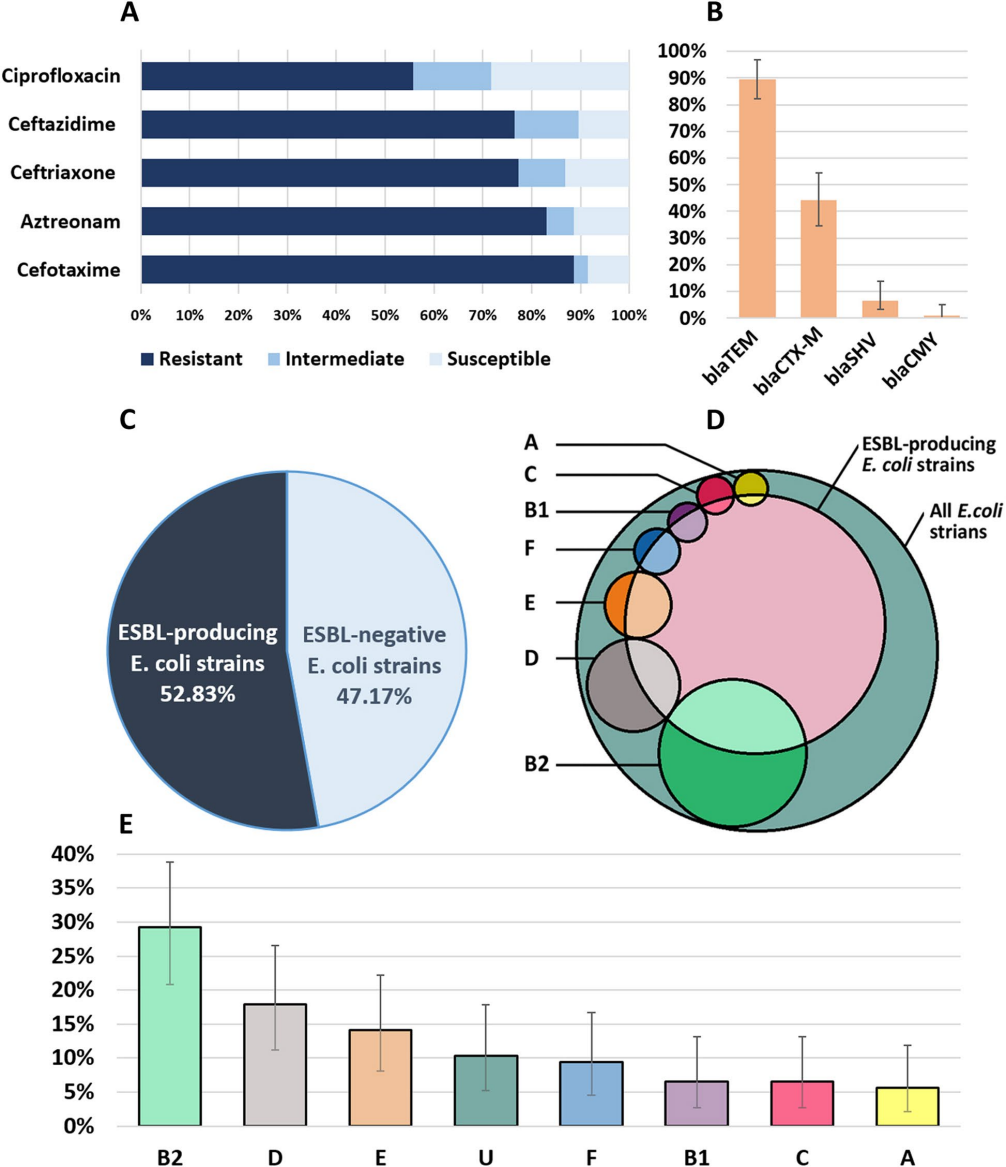
**A**: Prevaalence of resistant, intermediate and susceptible *E. coli* strains to studied antibiotics. **B**: Prevalence of antimicrobial resistant genes. **C:** Frequency of EABL-producing *E. coli* strains. **D**: The overlapping pattern of phylotypes with ESBL-positve and ESBL-negative* E. coli* strains. **E**: Frequency of phylotypes in this study

In this study, antibiotic resistance was assessed against six commonly used antibiotics in the treatment of urinary tract infections related to fluoroquinolones and β-lactam classes. In phenotypic tests, more than half of isolates were ESBL-producer (Fig. 1C). Only three isolates showed no phenotypic resistance against studied antibiotics (Table 1). Also, three isolates were resistant against just one antibiotic and the remaining (103 isolates; 97.1%, 95%CI: 91.9–99.9%) were resistant to more than one antibiotics (Table 2). The most common resistance pattern was related to CP/AZT/CRO/CAZ/CTX (Table 2). The results showed that the resistance against antibiotics cefotaxime and aztreonam were highly prevalent (Table 1 and Fig. 1A). However, ciprofloxacin showed the lowest rates of antibiotic resistance (Table 1 and Fig. 1A).

**Table 1:** Prevalence of phylogenetic groups among *E. coli* isolates and prevalence of AR genotypes and phenotypes among each phylotype

Variables	no. of isolates in each phylotype (% among 106 *E. coli* isolates)							
	A	B1	B2	C	D	E	F	U
	6 (5.6)	7 (6.6)	31 (29.2)	7 (6.6)	19 (17.9)	15 (14.1)	10 (9.4)	11 (10.3)
AR gene [no. (% among 106 *E. coli* isolates)]	Prevalence of AR gene; no. (% in each phylotype)							
* bla*_TEM_ [95 (89.6)]	6 (100)	6 (85.7)	25 (80.6)	7 (100)	18 (94.7)	15 (100)	10 (100)	8 (72.7)
* bla*_SHV_ [7 (6.6)]	-	-	1 (3.2)	-	1 (5.2)	1 (6.6)	-	4 (36.3)
* bla*_CTX-M_ [47 (44.3)]	2 (33.3)	3 (42.8)	15 (48.3)	4 (57.1)	6 (31.5)	8 (53.3)	6 (60)	3 (27.2)
* bla*_CMY_ [1 (0.9)]	-	-	1 (3.2)	-	-	-	-	-
Without AR gene [9 (8.4)]	-	1 (14.2)	5 (16.1)	-	-	-	-	3 (27.2)
AR phenotype [no. (% among 106 *E. coli* isolates)]	Prevalence of AR phenotype; no. (% in each phylotype)							
CP [59 (55.6)]	3 (50)	4 (57.1)	18 (57.6)	5 (71.4)	6 (31.5)	7 (46.6)	8 (80)	8 (72.7)
AZT [88 (83)]	6 (100)	6 (85.7)	26 (83.8)	7 (100)	13 (67.4)	11 (73.3)	9 (90)	10 (90.9)
CRO [82 (77)]	4 (66.6)	5 (71.4)	24 (77.4)	6 (87.7)	11 (57.8)	13 (86.6)	10 (100)	9 (81.8)
CAZ [81 (76.4)]	5(83.3)	4 (57.1)	25 (80.6)	7 (100)	12 (63.1)	10 (66.6)	10 (100)	8 (72.7)
CTX [94 (88.6)]	5 (83.3)	5 (71.4)	28 (90.3)	7 (100)	17 (89.4)	13 (86.6)	10 (100)	9 (81.8)
ESBL-producing strains [56 (52.8)]	2 (33.3)	5 (71.4)	14 (45.1)	3 (42.8)	9 (47.3)	10 (66.6)	8 (80)	5 (45.4)
Without AR phenotype [3 (2.8)]	-	1 (14.2)	-	-	1 (5.2)	1 (6.6)	-	-

**Table 2:** Frequency and distribution pattern of phenotypic AR profiles among phylotypes

Phenotypic resistance pattern	no. of isolates (%)	Phylotypes								ESBL-positive	ESBL-negative
		A	B1	B2	C	D	E	F	U		
CP, AZT, CRO, CAZ, CTX	42 (39.6)	2	2	14	4	2	5	8	5	28	14
AZT, CRO, CAZ, CTX	20 (18.8)	1	1	8	2	4	2	1	1	12	8
CP, AZT, CRO, CTX	3 (2.8)	-	-	1	-	1	-	-	1	1	2
CP, AZT, CAZ, CTX	3 (2.8)	1	-	-	1	1	-	-	-	-	3
CRO, CAZ, CTX	5 (4.7)	-	-	-	-	2	2	1	-	4	1
AZT, CAZ, CTX	5 (4.7)	1	-	1	-	1	-	-	2	2	3
AZT, CRO, CTX	6 (5.6)	-	-	1	-	2	3	-	-	3	3
CP, CAZ, CTX	2 (1.8)	-	-	-	-	1	1	-	-	1	1
CP, AZT, CRO	2 (1.8)	-	-	-	-	-	1	-	1	-	2
CP,AZT, CTX	1 (0.9)	-	1	-	-	-	-	-	-	1	-
CAZ, CTX	3 (2.8)	-	-	2	-	1	-	-	-	1	2
AZT, CTX	1 (0.9)	-	-	-	-	1	-	-	-	-	1
AZT, CRO	2 (1.8)	1	1	-	-	-	-	-	-	-	2
CP, CRO	1 (0.9)	-	-	-	-	-	-	-	1	-	1
CP, AZT	3 (2.8)	-	1	1	-	1	-	-	-	-	3
CTX	2 (1.8)	-	-	1	-	1	-	-	-	1	1
CP	2 (1.8)	-	-	2	-	-	-	-	-	-	2
No resistance	3 (2.8)	-	1	-		1	1			1	2
Total	106 (100)	6	7	31	7	19	15	10	11	55	51

### Prevalence of β-lactamase genes

**Fig. 2:**
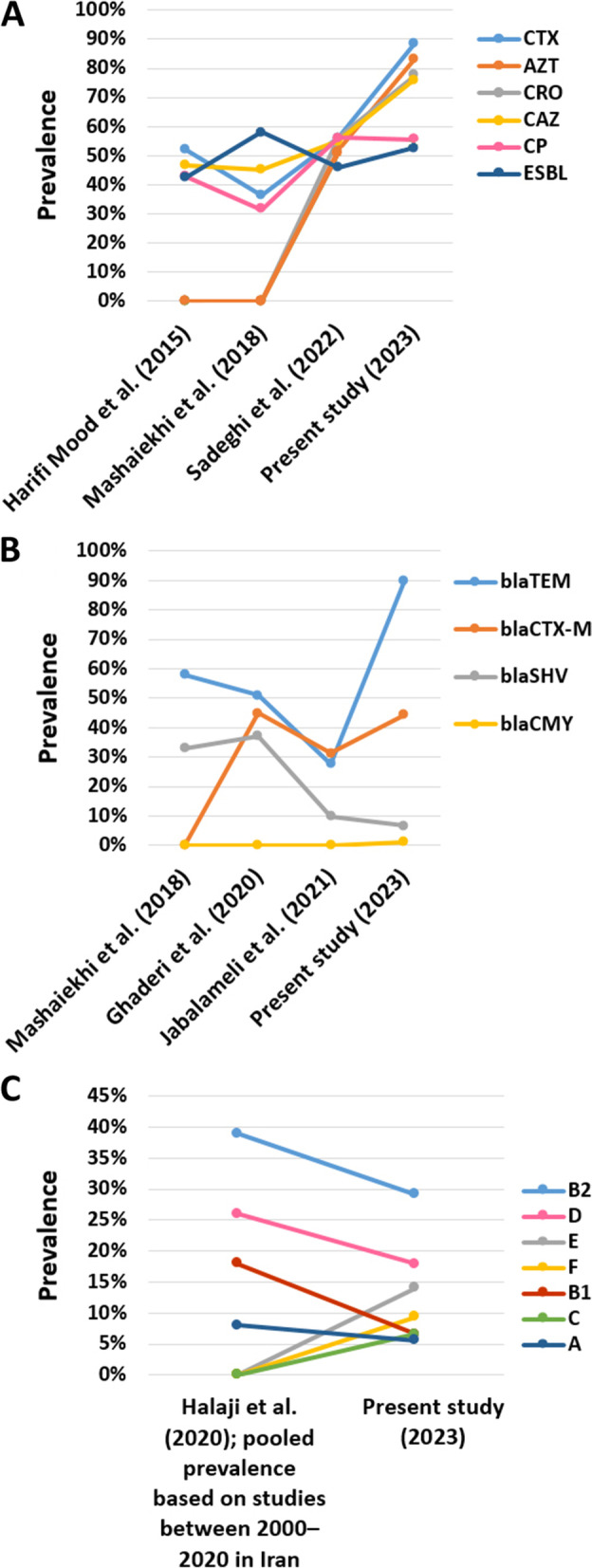
Comparison of phenotypic and genotypic characteristics between current research and some studies in different time periods in Iran. **A**: Prevalence of phenotypic antimicrobial resistant. **B**: Frequency of ARGs. **C**: Prevalence of phylotypes

In this study, among 106 isolates, *bla*_TEM_ was the most prevalent gene followed by *bla*_CTX-M_, *bla*_SHV_ and *bla*_CMY_, respectively (Table 1, Fig. 1B and Fig. 2). Also, the most prevalent resistance gene profiles were *bla*_TEM_ and *bla*_TEM_*/bla*_CTX-M_ (Table 3).

**Table 3:** Prevalence and distribution pattern of AR gene profiles among phylotypes

Resistance profile	Prevalence of profile (%)	phylotypes								ESBL-positive	ESBL-negative
		A	B1	B2	C	D	E	F	U		
*bla*_SHV_*/ bla*_TEM_*/bla*_CTX-M_	4 (3.7)	-	-	1	-	1	1	-	1	4	-
*bla*_TEM_*/bla*_CTX-M_*/bla*_CMY_	1 (0.9)	-	-	1	-	-	-	-	-	-	1
*bla*_TEM_*/bla*_CTX-M_	40 (37.7)	2	3	12	4	4	7	6	2	32	8
*bla*_TEM_*/bla*_SHV_	3 (2.8)	-	-	-	-	-	-	-	3	1	2
*bla*_CTX-M_	2 (1.8)	-	-	1	-	1	-	-	-	-	2
*bla*_TEM_	47 (44.3)	4	3	11	3	13	7	4	2	17	30
No resistance gene	9 (8.4)	-	1	5	-	-	-	-	3	2	7
Total	106 (100)	6	7	31	7	19	15	10	11	56	50

Odds ratio between AR genes and phenotypes significantly (*P* < 0.05) revealed that the isolates showing resistance against CTX, CAZ and CRO were 4.59, 3.24 and 8.1 times more likely to the positive for *bla*_CTX-M_ gene than the isolates which were susceptible to CTX, CAZ and CRO, respectively (Table 4). The ESBL-producing isolates were 24.95 and 6.38 times more likely (*P* < 0.05) to the positive for *bla*_TEM_ and *bla*_CTX-M_ genes, respectively, than the isolates which were negative for ESBL. Other odds ratios between AR genes and phenotypes were not significant (Table 4).

**Table 4:** Odds ratio between AR genes and phenotypes

Resistance gene	Phenotypic resistance					
	CTX	CAZ	CRO	AZT	CP	ESBL
*bla*_TEM_	1.88^a^	3	0.41	0.57	1.64	**24.95**^**b**^
*bla*_SHV_	2.14	1.92	4.86	3.4	5.2	2.35
*bla*_CTX-M_	**4.59**^**b**^	**3.24**^**b**^	**8.1**^**b**^	2.37	1.82	**6.38**^**b**^
*bla*_CMY_	0.4	0.95	0.09	0.06	0.26	0.29

^a^ The isolates showing resistance against cefotaxime (CTX) were 1.88 times more likely to the positive for *bla*_TEM_ gene than the isolates which were susceptible to CTX

^b^ Odds ratio with *P*-value < 0.05 is marked with letter "b" and are bold

### Prevalence of phylotypes

Overall, out of 106 *E. coli* isolates, phylotypes A, B1, B2, C, D, E, F and unknown (U) strains were identified; frequencies from high to low were B2 > D > E > F > B1 > C > A, respectively (Fig. 1D, 1E and 2). There was significant difference (*P* < 0.05) between B2 frequency and the other phylotypes.

## Discussion

ESBL-producing UPEC strains were widely reported in developing countries as one of the most critical challenges defined by World Health Organization (WHO) [15, 16]. More than half of our *E. coli* isolates were phenotypical ESBL-producer; this result is approximately similar to the prevalence of ESBL-producing *E. coli* isolates reported by Iranian studies during 2015–2022 [17, 18]. Our results are very similar to prevalence of ESBL-producing isolates in previous study in Jiroft (2018) [19]. Also, our results are similar to prevalence in China [20], higher than Nigeria [21] and comparable with a wide range of abundance in other African researches [22]. The initial treatment of UTIs with mainly β-lactam antibiotics may be responsible for the high rates of ESBL-producers in our study and other studies. During a surveillance program in Europe, *E. coli* isolates from UTIs in 18 countries were assessed, and almost one-fifth of all isolates were positive for ESBL phenotypes [23]. Various prevalence rates were reported in North America, showing the annual increase (approximately 8%) in ESBL-producing isolates [24, 25]. ESBL prevalence in this study was compared with some previous studies in Iran and other regions of the world in Table 5.

**Table 5:** Comparison of ESBL prevalence in this study with some studies in Iran and other regions of the world

Region	Year	Authors	Method	Sample size and origin	ESBL prevalence
Present study	2023	Afshari et al	Combined disk diffusion test	106 *E. coli* isolates from 168 UTI cases	52.8%
Iran (North)	2022	Sadeghi et al	Combined disk diffusion test	263 non-repetitive *E. coli* isolates from UTI cases	46%
Iran (Jiroft)	2018	Mashaiekhi et al	Combined disk diffusion test	181 *E.coli* isolates from UTI cases	58%
Iran (Northeast)	2015	Harifi Mood et al	Combined disk diffusion test	200 *E. coli* isolates from different clinical specimens	42.5%
China (Southwest)	2020	Sun et al	Vitek Compact 2 system	7713 non-repetitive UPEC isolates	49.7%
Europe	2020	Critchley et al	CLSI MIC screening	766 *E. coli* from UTI cases	17.9%
Africa (East)	2016	Sonda et al	Meta-analysis	4076 *Enterobacteriaceae* isolates	14%—89%
Nigeria	2012	Ejikeugwu et al	Double disk synergy test	83 non-repetitive *E. coli* isolates from suspected UTIs	27.7%
America (North)	2016	Lob et al	CLSI MIC screening	3498 *E. coli* isolates from UTI cases	7.8%—18.3%
USA	2021	Kaye et al	Retrospective study	1,513,882 *E. coli* isolates from UTI cases	9.2%

Many factors are involved in the emergence of ESBL-producing bacteria. Incorrect antibiotic use, self-medication, prescription without antimicrobial susceptibility tests, and consumption of counterfeit drugs may lead to selective pressure in favor of ESBL-producing bacteria and the detriment of susceptible strains to β-lactam antibiotics [26]. ESBL-producing strains could be transferred from one host to another and contaminate the environment at the same time. Therefore, admission to a hospital, surgery, hospitalization, a history of UTI, travel, and swimming are among the most significant risk factors for acquiring ESBL-producing *E. coli* strains [4, 16].

The prevalence of resistance against antibiotics ceftazidime, cefotaxime, aztreonam and ceftriaxone were considerably high. Approximately, it is in agreement with studies in Ethiopia, Nigeria and Cyprus [21, 27, 28] and higher than the results in Sri Lanka, US and Canada [23–25, 29, 30]. Furthermore, resistance towards cefotaxime was more frequent than against ceftazidime, in accordance with the results reported by Gaviria et al. (2022) in Cerdanya [30]. Frequency rate of resistance against ciprofloxacin was found in approximately half of our isolates. According to a retrospective observational study conducted in China, the resistance rate to ciprofloxacin in UPECs varies between 55 and 70% during 8-year, 2012 to 2019 [20]. The fluoroquinolone resistance level in our study was higher than some findings in Europe and North America [23, 24]. Prevalence of phenotypic antimicrobial resistance in this study was compared with some previous studies in Iran and other regions of the world in Table 6. The most common pattern of resistance in our study was related to all six studied antibiotics, including CP/AZT/CRO/CAZ/CTX. This profile is not considered a multidrug-resistant (MDR) pattern because we studied only two antimicrobial categories; MDR means non-susceptibility to at least one agent in three or more antimicrobial classes. Nevertheless, resistance to more than one antimicrobial agent leads to problems in treatment of infectious disease.

**Table 6:** Comparison of phenotypic antimicrobial resistance prevalence in this study with some studies in Iran and other regions of the world

Region	Year	Authors	Method	Sample size and origin	CTX	CAZ	CRO	AZT	CP
This study	2023	Afshari et al	Disk diffusion test	106 *E. coli* isolates from 168 UTI cases	88.6%	76.4%	77%	83%	55.6%
Iran	2022	Sadeghi et al	Disk diffusion test	263 *E. coli* isolates from UTI cases	NA	55.1%	56.3%	52.1%	56.3%
Iran (Jiroft)	2018	Mashaiekhi et al	Disk diffusion test	181 *E.coli* isolates from UTI cases	36.4%	45.3%	NS	NS	31.4%
Iran	2015	Harifi Mood et al	Disk diffusion test	200 *E. coli* isolates from clinical specimens	52%	46.5%	NS	NS	43%
China	2020	Sun et al	Vitek Compact 2 system	7713 non-repetitive UPEC isolates	NS	26.1%	54%	39.6%	61.8%
Ethiopia	2020	Dadi et al	Disk diffusion test	200 *E. coli* isolates from 780 UTI cases	66%	84%	80.5%	NS	14.5%
Nigeria	2012	Ejikeugwu et al	Disk diffusion test	83 *E. coli* isolates from suspected UTI cases	76%	64%	34%	NS	47%
Spain	2022	Gaviria et al	MIC	26 *E. coli* isolates from 30 UTI cases	100%	53.8%	NS	NS	80.7%
Cyprus	2016	Cantas et al	BD Phoenix ™	389 *E. coli* isolates from Cystitis cases	NS	75%^a^	80%^a^	90%^a^	90%^a^
USA	2019	Critchley et al	MIC	1831 *E*. *coli* isolates from UTI cases	NS	8.5%	NS	NS	25.8%
USA	2016	Lob et al	MIC	3498 *E. coli* isolates from UTI cases	19.3%	16.8%	19.5%	NS	35.3%
Canada	2016	Lob et al	MIC	3498 *E. coli* isolates from UTI cases	14.8%	8.3%	14.3%	NS	25.5%

^a^ Approximately; *NS* Not studied, *NA* no access

Resistance to β-lactams and fluoroquinolones limits options in the treatment of UTI associated with ESBL-producing *E. coli*. Therefore, choosing the best antibiotic should be based on the severity of UTI and antibiogram tests. Nevertheless, there are some suggestions for treatment of resistant UTIs including piperacillin-tazobactam, meropenem/vaborbactam, imipenem/cilastatin-relebactam, ceftazidime-avibactam, ceftolozane-tazobactam, plazomicin, cefiderocol, fosfomycin, sitafloxacin, finafloxacin, colistin, and tigecycline [31].

The use of antibiotics in veterinary medicine for food-producing animals is an important reason for the development of resistance in *E. coli* as a member of gut microbiota. It can lead to two phenomena: 1) The emergence of resistant bacteria in animals and transmission of them through the food chain or direct contact. 2) Transmission of antimicrobial agents to humans through animal products containing antibiotic residues and subsequently emergence of resistant bacteria in humans [32]. Since antibiotics such as bacitracin, virginiamycin, colistin, etc. may be used as growth promoters in food-producing animals, the products must be tested to check the antibiotic residue according to the laws in Iran.

In level of antimicrobial resistance genes (ARGs), β-lactamase (*bla*) genes were studied; the results showed that *bla*_TEM_ and *bla*_CTX-M_ genes were positive with a high prevalence rate, but *bla*_SHV_ and *bla*_CMY_ had low frequency. Meta-analytic studies showed that the prevalence of ESBL genes was significantly high in different regions of Iran with various rates [33, 34]. Similar to our study, high frequencies of *bla*_TEM_ and *bla*_CTX-M-_positive isolates were reported in other countries and regions, such as northern and eastern Europe [35]. In many countries, *bla*_CTX-M_ group genes are prevailed, and rapidly disseminating among different *Enterobacteriaceae* causing UTIs [36]; for example, the rate of this gene was evaluated more than 85% in *E. coli*–causing bloodstream and urinary tract infections in patients hospitalized in the US [37]. The results of the present study on the genes *bla*_TEM_ and *bla*_CTX-M_ were higher than some studies in South Africa and Nigeria [38, 39]. The prevalence of *bla*_SHV_ was near to the results of Sri Lanka [29]. Prevalence of *bla*_CMY_, one of the most common plasmid-mediated AmpC β-lactamase gene, was lower than Sri Lanka [29], and close to the findings in China [40]. ARGs prevalence in this study was compared with some previous studies in Iran and other regions of the world in Table 7.*bla* genes were originally chromosomal which is incorporated into plasmid and has spread to various *Enterobacteriaceae* members. These genes usually acquired by the horizontal gene transfer from other bacteria using mobile genetic elements such as conjugative plasmids or transposons [22]. *bla* genes have been detected in hospitals and clinics worldwide and are often responsible for resistance phenotypes to β-lactam antibiotics [41]. *bla*_TEM_ is one of the most well-known determinants of resistance with more than 170 variants. It seems that *bla*_SHV_, *bla*_CTX-M_ and *bla*_OXA_ genes are mutants of classical *bla*_TEM_ genes [41]; *bla*_TEM_ was significantly found in ESBL producing strains of present work (Table 4). Also, a significant relationship was found between the presence of the *bla*_CTX-M_ gene and the resistance phenotype against the antibiotics ceftazidime, cefotaxime and ceftriaxone (Table 4) which is in agreement with previous researches; *bla*_CTX-M_ encodes enzymes for the hydrolysis of cephalothin, cephaloridine, penicillin, cefotaxime and ceftazidime [42]. Previous studies show that *bla*_SHV_ is responsible for resistance to penicillins such as ampicillin and piperacillin [42]. *bla*_CMY_ is a type of AmpC plasmid that may cause antibiotic resistance to ceftazidime, cefotaxime, cefoxitin, azetronam and probably cefepime [43].

**Table 7:** Comparison of ARGs prevalence in this study with some studies in Iran and other regions of the world

Region	Year	Authors	Method	Sample size and origin	*bla*_TEM_	*bla*_CTX-M_	*bla*_SHV_	*bla*_CMY_
This study	2023	Afshari et al	PCR	106 *E. coli* isolates from 168 UTI cases	89.6%	44.3%	6.6%	0.94%
Iran (Jiroft)	2018	Mashaiekhi et al	PCR	181 *E.coli* isolates from UTI cases	58%	NS	33%	NS
Iran	2020	Ghaderi et al	Systematic review	68 studies on *E. coli* isolates from clinical cases	51%	45%	37%	NS
Iran	2021	Jabalameli et al	Systematic review	61 studies on *E. coli* isolates from clinical cases	27.6%	31.2%	9.8%	NS
China	2021	Jia	PCR	332 ESBL-producing and -uncertain *E. coli* isolates	NA	NA	NA	0.3%
Sri Lanka	2022	Perera et al	PCR	422 *Enterobacteriaceae* isolates from UTIs	25%	33%	8%	15%
South Africa	2020	Kubone et al	PCR	26 *E. coli* isolates from 143 urine samples	3.84%	11.53%	0%	NS
Africa (East)	2016	Sonda et al	Meta-analysis	4076 *Enterobacteriaceae* isolates	16–55%	70–88.5%	3–64%	NA
Nigeria	2016	Mohammed et al	PCR	439 *E. coli* isolates from clinical specimens	31.4%	27.3%	36.4%	NS
Europe	2019	Sepp et al	WGS	10,780 *E.coli* isolates from clinical cases	54.9%	77.5%	21.4%	7.9%
US	2019	Mendes et al	NGS	2751 *E. coli* isolates from blood and UTI cases	0.3%	87.6%	0.3%	7.8%
USA	2016	Lob et al	Microarray and PCR	181 ESBL-positive *E. coli* isolates from UTI cases	3.3%	91.7%	2.2%	NA
Canada	2016	Lob et al	Microarray and PCR	74 ESBL-positive *E. coli* isolates from UTI cases	1.5%	87.8%	2.9%	NA

*NS* Not studied, *NA* no access, *WGS* whole genome sequencing, *NGS* next generation sequencing

*E. coli*, as one the main member of commensal papulation in the intestinal microflora, have stable genetic structure with a moderate levels of recombination in their genome. This genetic trait result in a clonal status in bacterial population and this is considered as a principle for identification of strong phylogenetic groups [44]. Today, four prevalent phylotype, including A, B1, B2, and D and four scarce phylotypes including C, E, F, and G have identified for *E. coli* bacterium. Some phenotypic and genotypic differences, including antibiotic resistance, virulence factors and growth rate, have introduced among various phylotypes [45]. For example, strains of UPEC pathotype usually belong to phylotypes B2 and D, but intestinal pathogenic and commensal *E. coli* strains belong to A and B1 [45]. In the present study, the most common phylotypes were B2 and D which is in agreement with other works in Iran [46] and Ethiopia [27]. Nevertheless, Mohsin et al. (2022) reported interesting results that differed from many studies; phylotyping of *E. coli* isolates from 500 UTIs in Iraq showed that the most frequent phylotype was F, followed by C > B2 > E > A > D > B1, respectively [47]. Table 8 compares the prevalence of phylotypes in our study with that of previous studies conducted in Iran and other regions of the world. However, it should be noted that there are numerous studies on this topic conducted in different parts of the world that were not included in our analysis.

**Table 8:** Comparison of phylotype frequencies in this study with some studies in Iran and other regions of the world

Region	Year	Authors	Method	Sample size and origin	A	B1	B2	C	D	E	F	U
This study	2023	Afshari et al	PCR	106 *E. coli* isolates from 168 UTI cases	5.6%	6.6%	29.2%	6.6%	17.9%	14.1%	9.4%	10.3%
Iran	2022	Halaji et al	Meta-analysis	68 studies on UPEC strains	8%	18%	39%	NS	26%	NS	NS	NS
World	2017	Stoppe et al	Meta-analysis	Commensal isolates in worldwide studies	36.1%	16.4%	20%	21.5%	NS	NS	NS	NS
Iraq	2022	Mohsin et al	PCR	118 isolates of *E. coli* from UTI cases	3.4%	1.7%	15.3%	20.3%	3.4%	14.4%	37.3%	4.2%
Ethiopia	2020	Dadi et al	PCR	200 *E. coli* isolates from 780 UTI cases	18.5%	24%	30%	0%	27.5%	0%	0%	0%
Australia	2020	Touchon et al	Pan-genome	5100 human and non-human samples	24%	24%	25%	NA	14%	NA	NA	NA

*NS* Not studied, *NA* no access

Halaji et al. (2022) reported an increasing trend for phylotype B2 incidence from 2014 to 2020 among UPEC infections during a meta-analytic systematic review; they have introduced several variables including host species, nutrition types, infection types, geographical regions, methodology, sample size and time of study to explain the variation in phylotype frequency in different researches [46]. Moreover, Touchon et al. (2020) introduced some factors, such as host (species, diet, sex, age and body mass), environment (climate and geographic location) and bacteria (resistance and virulence) for the distribution pattern of phylotypes [45].

There were several limitations and weaknesses in this study, such as the small sample size, the limited number of regions and cities examined, and the absence of patient history, including information on prior infections, recurrent UTIs, and antibiotic use. Therefore, we suggest conducting further studies, such as a survey on antibiotic-resistant *E. coli* isolates from fecal samples of UTI cases in multiple cities, to provide a comprehensive overview of the antimicrobial resistance prevalence at the national level.

## Conclusions

This work shows the high prevalence of ESBL-producing *E. coli* strains and the predominance of *bla*_*TEM*_ and *bla*_*CTX*-*M*_ genes among the strains that mostly belonged to the pathogenic phylotypes B2 and D. Best of our knowledge, *bla*_CMY_ gene and the phylotypes C, E and F were reported for the first time in Jiroft in this study. Comparison of our findings with some previous studies in different time periods in Iran shows the increasing trend of phenotypic and genotypic antimicrobial resistance prevalence in Iran and Jiroft city (Fig. 2); therefore, this study highlights the need for urgent action to prevent the spread of antibiotic resistance, such as strict rules to control the sale of antibiotics and presence of antibiotic residues in foods produced by animals. This study emphasizes the urgent need for awareness about antibiotic use in the community and implementation of a national surveillance system to monitor antibiotic-resistant bacteria.

## Data Availability

All data generated or analysed during this study are included in this published article. Also, our dataset does not include proteomics data and protein sequences, DNA and RNA sequences, genetic polymorphisms, linked genotype and phenotype data, macromolecular structure, gene expression data, and crystallographic data for small molecules. So, our data does not fall under the list of data types that must be deposited in BMC recommended repositories.
